# A Synoviocyte Model for Osteoarthritis and Rheumatoid Arthritis: Response to Ibuprofen, Betamethasone, and Ginger Extract—A Cross-Sectional *In Vitro* Study

**DOI:** 10.1155/2012/505842

**Published:** 2012-12-31

**Authors:** Søren Ribel-Madsen, Else Marie Bartels, Anders Stockmarr, Arne Borgwardt, Claus Cornett, Bente Danneskiold-Samsøe, Henning Bliddal

**Affiliations:** ^1^Department of Rheumatology, The Parker Institute, Copenhagen University Hospital Bispebjerg and Frederiksberg, Nordre Fasanvej 57, 2000 Frederiksberg, Denmark; ^2^Institute for Informatics and Mathematical Modelling, Technical University of Denmark, 2800 Lyngby, Denmark; ^3^Department of Orthopaedic Surgery, Copenhagen University Hospital Bispebjerg and Frederiksberg, 2000 Frederiksberg, Denmark; ^4^School of Pharmaceutical Sciences, University of Copenhagen, Universitetsparken 2, 2100 København Ø, Denmark; ^5^Faculty of Health Science, University of Copenhagen, 2200 Copenhagen N, Denmark; ^6^Center for Sensory-Motor Interaction, Aalborg University, 9220 Aalborg, Denmark

## Abstract

This study aimed at determining if synovial cell cultures from rheumatoid arthritis (RA), osteoarthritis (OA), and healthy controls (HC) differ and are suitable disease models in pharmacological studies, and tested their response to some anti-inflammatory drugs. Synovial cells were isolated from synovial membrane or joint fluid. Cells were cultivated and exposed to no or TNF-**α** stimulation without, or in the presence of, betamethasone, ibuprofen, or a standardized ginger extract. Concentrations of a panel of cytokines, growth factors, and chemokines were mapped for each culture and condition. Our cells secreted an increased amount of the cytokines IL-1**β**, IL-6, and IL-8 in response to TNF-**α** stimulation in all conditions. OA cells showed a higher IL-6 and IL-8 and a lower IL-1**β** production, when not stimulated, than RA and HC cells, which were similar. TNF-**α** stimulation caused similar IL-1**β**, IL-6, and IL-8 release in all groups. Ibuprofen showed no effect on cytokine production, while ginger extract was similar to betamethasone. Ginger extract was as effective an anti-inflammatory agent as betamethasone in this *in vitro* model. Cultured fibroblast-like synoviocytes from OA and RA subjects promise to be a useful pharmacological disease model, but further studies, to support results from such a model are needed.

## 1. Introduction

Rheumatoid arthritis (RA) is considered a systemic disease with a variety of determinants for its pathogenesis, including T-cell and B-cell dependent pathways [1]. Inflammatory and autoimmune processes interact with nonimmune cell types, resulting in cartilage and bone attack [2]. The finding that fibroblast-like synoviocytes from RA patients can invade cartilage and destruct it, and that these synoviocytes maintain these characteristics over a period of time in an *in vivo* animal experiment in the absence of T-cells [3] indicates an involvement of fibroblast-like synoviocytes in the inflammatory response in RA. The possible activation of fibroblast-like synoviocytes in patients with osteoarthritis (OA) has been far less considered. It is believed that the balance between anabolic and catabolic processes in healthy cartilage is driven by cytokines, and an excess of proinflammatory cytokines is thought to result in many of the clinical manifestations of OA [4]. The expression of some cytokines and matrix-degrading enzymes has been compared between cultured fibroblast-like synoviocytes from RA and OA, showing far more proliferation and cytokine production in RA [5]. Demonstration and rating of some cytokines and mononuclear cell infiltrates by immunohistochemistry examination of synovial membrane biopsies from RA and OA patients have shown higher concentrations in RA, although all the cytokines looked for were present in both groups [6].

The cells present in the synovial membrane were first classified as types “A” and “B” [7] and later described as macrophage-like synoviocytes (type A lining cells) and fibroblast-like synoviocytes (type B lining cells), respectively [8]. The synovial membrane has also endothelial cells which line the lumina of blood vessels and interact with immune cells in the blood, mainly leukocytes, resulting in extravasation of these cells, and this is important in the pathogenesis of inflammatory disorders [9]. All of these four cell types respond to cytokines and produce cytokines themselves. It has been stated that molecular and functional characteristics of cultures of adherent, mostly, fibroblast-like synoviocytes can provide evidence for pathogenic mechanisms [10]; hence these characteristics are conserved under *in vitro* culture. 

We have considered if it was possible to establish an *in vitro* model of the synovial membrane and cavity for studies on the conversion of prodrugs intended for topical administration into the joint to the pharmacologically active substance. For simplicity and feasibility, this model was made up from adherent cells cultured from synovial fluid or synovial membrane biopsies, and thus devoid of T- and B-cells, and consequently with absence of important interactions that occur *in vivo*. Other objectives were to identify products from the synovial cells contributing to the inflammation, which may be used as markers of OA and RA and possibly distinguishing these diseases, and to identify combined profiles of cytokines characteristics of OA or RA as compared to a constitutional production by cells from noninflamed tissue. 

The aim of the present study was therefore, by mapping the concentrations of a panel of cytokines and chemokines, with no stimulation and after stimulation with tumour necrosis factor *α* (TNF-*α*), to see if the cell cultures differ between diagnosis of the donor and differ compared to healthy control cells, and if the treatments with betamethasone, ibuprofen, and a standardized extract of the herbs *Zingiber officinale* and *Alpinia galanga,* with known anti-inflammatory substances, show any differences in cell reactions.

## 2. Materials and Methods

### 2.1. Ethics

The study was accepted by Copenhagen and Frederiksberg Municipalities Ethics Committee (Protocol: Metabonomics-KF-01255092).

### 2.2. Samples

Samples were taken from OA and RA patients, as well as from healthy controls. The OA and RA diagnoses of the included patients were according to the ACR criteria [11–13]. 

The samples of synovial fluid were aspirated from the joint cavity under ultrasound echography guidance, and the synovial membrane specimens from OA or RA patients, approximately 5 g, were taken in connection with orthopaedic surgery. 

The samples from healthy controls (HC) were biopsies of the synovial membrane, five specimens of approximately 10 mm^3^ each, taken from the knee with forceps through an endoscope, from subjects who had one knee endoscopically examined for meniscus injury and had consented to have biopsies taken from the opposite knee, which had given no symptoms of inflammation like pain or malfunction. None of these healthy controls had any pre-history of, or showed any sign of, arthritis.

### 2.3. Isolation and Cultivation of Synovial Cell

Synovial cells were isolated from either biopsies of synovial membrane or samples of joint fluid. Tissue biopsies were minced into pieces with a length of 3 mm along their longest axis. The amount of joint fluid that had been taken, ranging from 0.5 to 50 mL, was centrifuged at RCF 500 ×g at 20°C, and the sediment was transferred to a T-25 tissue culture flask containing DMEM/high glucose reduced serum (HyClone, Cat No. SH30565, Thermo Scientific) with 8% Fetalclone III foetal bovine serum (FBS) (HyClone, Cat No. SH30109, Thermo Scientific), penicillin 60 i.u./mL, and streptomycin 60 *μ*g/mL. The flasks were placed in a humidified atmosphere with 5% carbon dioxide at 37°C, and the medium was entirely replaced with fresh medium with intervals of seven days. The adherent cells were harvested after detachment with trypsin and EDTA when the culture had reached 75% confluence, frozen-in in FBS with 10% dimethylsulfoxide at a cooling rate of 1°C/min, and stored in liquid nitrogen. 

For the experiments, cryopreserved cell isolates which had been passed 1 or 2 times before they were frozen were selected. These cryo-preserved cell isolates were rapidly thawed and gently mixed with the previously mentioned medium, grown to 75% confluence, detached, split, replated, and again grown until a minimum of 150 cm^2^ flask bottom area was covered with a 90% confluent layer of cells with good fibroblast morphology, corresponding to minimum 1.5 × 10^7^ cells. This propagation required four to six passages and at the same time secured total wash-out of any drugs used by the patients.

A sample from the medium, conditioned through three days' culture, was subjected to an RNA hybridization assay, which is able to demonstrate infection with any of the eight *Mycoplasma *species accounting for approximately 95 percent of all instances of *Mycoplasma *contamination (MycoProbe, R&D Systems, Minneapolis, MN, USA). All cell cultures used for the experiments in the present study were tested negative for *Mycoplasma *contamination, like by far most cell cultures tested routinely in our laboratory.

The cells in the cell culture flasks used for multiplication of a given cell isolate were detached with Dulbecco's phosphate buffered saline (DPBS) containing 1% bovine serum albumin (BSA) and 2 mM EDTA by standing at 4°C for 10 minutes and occasional knocking.

### 2.4. Presentation of the Synovial Model

The practical presentation of the synovial model was as follows: cells grown adherent to the bottom of cell culture plates, each plate divided into eight square fields with an area of 8.6 cm^2^ each, and two rows by four columns (Nunc, Cat No. 176600, Denmark). For each cell population were used a minimum of three rows by four fields each, in 14 experiments six rows and in five experiments nine rows. The rows were designated “A,” “B,” and “C,” respectively. One field in each row was used for the control treatment with no pharmacologically active substance added, and the other three fields were used for treatment with anti-inflammatory substances, to be reported separately. The suspension of cells that had been multiplied and harvested was centrifuged at RCF 300 ×g for 10 minutes. Then the cell precipitate was resuspended in DMEM/high glucose reduced serum with 3% FBS, penicillin 60 i.u./mL, and streptomycin 60 *μ*g/mL, 1.0 mL per field. The cell population concerned was intended for plus 1.0 mL excess. To each field of the eight-field plates was first pipetted 1.0 mL DMEM/high glucose reduced serum with 3% FBS, followed by 1.0 mL of the cell suspension. This favoured a uniform distribution. The cell culture plates were placed in humidified atmosphere with 5% carbon dioxide at 37°C for two hours, then the medium was replaced with 2.0 mL medium per field, and the plates were incubated. 

### 2.5. Analysis of Composition of Cell Populations Used

The composition of each cell population, dispensed into eight-well cell-culture plates, and used for a substance exposure experiment, was characterised in connection with the plating. The suspensions of harvested cells from several flasks were pooled, centrifuged at RCF 300 ×g for 10 minutes, the supernatant was aspirated, and the cells were resuspended in 5 mL DPBS with 1% BSA. Aliquots at 50 *μ*L each were distributed to ten tubes, and these separate aliquots of the cells were stained with antibodies to CD14, which is expressed on macrophages including macrophage-like synoviocytes (clone M5E2, RPE-conjugated, BD Biosciences Pharmingen, San Diego, CA, USA), with CD31, which is expressed on endothelial cells, macrophages, and some cells that were unlikely to be present (clone WM59, RPE-conjugated, BioLegend, San Diego, CA, USA), or with CD90, also called Thy1, which is expressed on fibroblasts including fibroblast-like synovial cells at least from the subintimal region of the synovial membrane [14], activated endothelial cells, and some cells that were unlikely to be present, and, importantly, CD90 is not expressed on macrophages (clone 5E10, APC-conjugated, BD Biosciences Pharmingen, San Diego, CA, USA). To three other aliquots of the cells were added mouse IgG of the same isotype as the antigen-specific antibody and conjugated with the same fluorochrome (from BioLegend, San Diego, CA, USA), to serve as isotype-matched control cells in the subsequent analysis by flow cytometry. One aliquot of cells was stained with anti-CD90 and then fixed with 1% formaldehyde, permeabilized with 0.027% saponine, and then stained with antibodies to CD68, which is expressed in cytoplasmic granules of macrophages, and in some cells that were unlikely to be present, but not expressed in fibroblasts (clone Y1/82A, RPE -conjugated, BD Biosciences Pharmingen, San Diego, CA, USA), and another tube of fixed and permeabilized cells was labelled with isotype- and fluorochrome-matched antibodies to the anti-CD68 and anti-CD90 used. One tube of cells was left unstained, and one tube was fixed with formaldehyde but not stained, for use as unstained control for anti-CD68. All labelings of cells with the fluorochrome-conjugated antibodies were done in DPBS with 1% BSA, with formaldehyde or saponine added when staining with anti-CD68, standing at 4°C for 40 minutes. The concentrations used were those recommended by the manufacturer. The antibody-stained cells were washed once, resuspended in 90 *μ*L DPBS with 1% BSA, transferred to microplate wells, and analyzed in a FACSArray Bioanalyzer flow cytometer (Becton Dickinson Immunocytometry Systems, San José, CA, USA). In the analysis of data, the gate for cells counted “positive” according to a marker was set on the isotype control histogram, referring to a marker concerned to include no or a few cells, maximum 3%, after which the number and median fluorescence intensity (MFI) of the marker-positive cells in this gate were recorded. 

### 2.6. Test Substances

Three test substances or preparations were chosen, aiming at representing a range of pharmacological mechanisms of action: betamethasone, ibuprofen, and an extract designated “EV.EXT77” (Ferrosan, Denmark) that had been produced by extraction of the rhizomes of the herbs *Zingiber officinale* and *Alpinia galanga*, both of which belong to the ginger family. The extract EV.EXT77 is standardized as to its concentration of anti-inflammatory substances.

Betamethasone has several mechanisms of action; a recent review deals with gene regulation by glucocorticoids [15]. Major mechanisms are inhibition of transcription factors AP-1 and NF-*κ*B, which in noninflamed conditions activate the genes for cyclooxygenase-2 (COX-2), some cytokines, and inducible NO synthase (iNOS). COX-2 (and its isoenzyme COX-1) increases the production of prostanoids (prostaglandins, thromboxanes, and prostacyclins, all of which have type members that mediate the inflammatory process), resulting in reduced inflammation. Another major mechanism is modification of basic and induced transcription of genes for collagenase. Hence the effects of betamethasone are reduced production of prostanoids due to the reduced *COX-2*-expression, reduced production of the proinflammatory cytokines and chemokines, and reduced production of iNOS. Ibuprofen is a nonsteroidal anti-inflammatory drug (NSAID) [16] and is a nonselective inhibitor of both isoenzymes COX-1 and COX-2, which catalyze the production of prostaglandins and thromboxanes. COX-2 is almost exclusively expressed on sites of inflammation; hence the action of ibuprofen appears as a reduced intensity of the inflammatory process. In addition, ibuprofen inhibits the enzyme 5-lipoxygenase (LOX) at the concentration used in our study [17]. LOX catalyzes the synthesis of leukotrienes from arachidonic acid, which is also the starting substance for the synthesis of prostaglandins and thromboxanes. Leukotrienes are important mediators in inflammatory processes. Ibuprofen also inhibits the bradykinin and histamine inflammatory pathways [18].

 The known anti-inflammatory substances in ginger are gingerols, shogaols, or paradols, all of which consist of series with 4, 6, or 8 methylene groups in a side chain of the molecule [19]. Several of the members of these three groups of substances inhibit COX-1 and COX-2, as do ibuprofen and other NSAIDs, and in addition substances from ginger will inhibit the enzyme 5-lipoxygenase (LOX) [19].

Betamethasone was applied as an aqueous solution of betamethasone 21-phosphate disodium (Sigma, St. Paul, MO, USA) with concentration of 1.32 mg/mL, equivalent to betamethasone-base 1.00 mg/mL. Ibuprofen (Sigma) was dissolved in water to a concentration of 1.00 mg/mL in a water bath at 100°C for five minutes. The fluid ginger extract EV.77/15 (Ferrosan A/S, Denmark) was made up to a concentration of 1.00 mg/mL by dissolving the extract in water for five minutes on a boiling water bath for five minutes during regular shaking. The concentration of the extract was chosen according to Grzanna et al. [19] and as recommended by the firm. The three solutions were added to DMEM medium with 3% FBS, penicillin, and streptomycin in volumes 1+9, such that the concentration of betamethasone-base, ibuprofen, or ginger extract 77/15 was 100 *μ*g/mL in the cell culture medium. As control solution (no treatment), a mixture of sterile water to DMEM medium with 3% FBS, penicillin, and streptomycin, 1+9 (volume) was used. The final media with additions were filtered through an 0.22 *μ*m membrane filter. 

### 2.7. Time Course of Cell Experiments

The eight-field plates were inspected in an inverse microscope after their cells, following seeding, had been cultured for 2 or 3 days, and the degree of confluency and the appearance of the cell layer were recorded. Only fields containing cells with good fibroblast morphology and not less than 40% confluency were taken for continuation of the experiment. The used cell-culture medium was aspirated completely and replaced with 1.5 mL of either DMEM/high glucose reduced serum with 3% Fetalclone III, diluted 9 parts of the medium with one part of sterile water for the untreated control group, or diluted in the same ratio with solutions of anti-inflammatory substances. The cell plates were then cultured for 72 hours, inspected as mentioned previously, and the cell-culture medium was aspirated completely. These samples of conditioned medium were aliquoted to labelled cryostorage tubes and stored at −80°C until analysis. The cells in the four fields in rows “A” were harvested after they had been rinsed with 1.0 mL phosphate-buffered saline under 2 minutes shaking, detached with phosphate-buffered saline with 0.5 mM EDTA, scraped off with a cell scraper, transferred almost completely to a microcentrifuge tube, and pelleted in this for later lysing aiming at analysis of cytosolic substances. 

To the rows “B” of the cell plates were added 1.5 mL of a freshly prepared portion of either DMEM/high glucose reduced serum with 3% Fetalclone III, diluted 9 : 1 with sterile water or with solutions of anti-inflammatory substances, and to the rows “C” were added 1.5 mL of the same media for untreated control or with test substances added, but with addion of TNF-*α* 1 nanogram per mL.

### 2.8. Quantitative Determination of Cytokines and Chemokines

The concentrations of the following cytokines and chemokines in the samples of conditioned cell culture media were determined by enzyme-linked immunosorbent assay (ELISA): chemokine (C-C motif) ligand 2 (CCL2, also referred to as MCP-1), interleukin-10 (IL-10), and macrophage migration inhibitory factor (MIF), or by multiplex-immunosorbent assay (MIA): granulocyte-macrophage colony-stimulating factor (GM-CSF), interferon-*γ* (IFN-*γ*), interleukin-1*β* (IL-1*β*), interleukin-6 (IL-6), interleukin-8 (IL-8) also referred to as CXCL8, interleukin-11 (IL-11), interleukin-12p70 (IL-12p70), interleukin-15 (IL-15), and interleukin-17 (IL-17). The ELISA or MIA determinations were performed with capture antibodies, detection antibodies, and recombinant human protein as calibrator standard, all from R&D Systems, Minneapolis, MN, USA. The reagents for MIA determinations were prepared in our own laboratory: capture antibodies were covalently coupled to fluorescent, carboxylated beads (Luminex, Austin, TX, USA) as devised [20, 21]. The concentrations of capture antibodies in the coupling step had been titrated in numerous experiments. The density of the combined suspension of these capture antibody beads was adjusted to the amount intended for each sample, 1500 beads in a volume of 25 *μ*L. Aliquots of 25 *μ*L each of the media samples were pipetted to the wells of a microplate and incubated with the capture antibody beads for 1.5 hours with agitation, then a solution of the detection antibodies for all analytes was added, and incubation resumed for 2 hours, the beads were washed twice with phosphate-buffered saline with intervening agitation for 5 minutes, centrifugated for pelleting of the beads at the bottom of the wells, and the supernatant was aspirated by a microtiter plate washer. A solution of streptavidine RPE (Invitrogen, Molecular Probes, Eugene, OR, USA) for coupling of this fluorochrome reporter to the detection antibodies was added, the bead suspension in the microplate wells was incubated for 30 minutes, the beads were washed twice, resuspended in sheath fluid for flow cytometry, and analyzed on the BD FACSArray flow cytometer. The data were analyzed by SigmaPlot 11 (Systat Software, San José, CA, USA), using 4-parameter logistic regression for expression of calibration curves and then calculation of sample concentrations with these parameter values and correction for recovery determined in the same assays.

### 2.9. Quantitative Determination of Histamine and Hydroxyproline

The concentrations of free hydroxyproline and histamine in the media samples were determined as devised [22]. Following derivatization of 30 *μ*L-aliquots with 6-aminoquinolyl-N-hydroxysuccinimidyl carbamate, separation of the sample components was carried out by high performance liquid chromatography using a Kinetex 2.6 *μ*m C18 150 × 4.6 mm column (Phenomenex, Torrance, CA, USA), in an HPLC instrument from Waters (Milford, MA, USA) with fluorescence monitoring of the column eluate. The method is intended for amino acid analysis, but measures histamine as well.

### 2.10. Statistical Methods

The data on concentrations of cytokines and chemokines were analyzed in a variance component model [23] with cell culture ID as a random component and fixed effects of diagnosis, treatment, whether cells were stimulated with TNF-*α*, sample location (elbow, finger or hand, hip, or knee), sample type (synovial fluid or synovial membrane), and age and sex of patients. The basic model (given for IL-6) was then

(1) ln⁡⁡(IL-6ij)=βDiagnosis, Stimulation, Treatment+βSpltype +βLocalization+βSex+βAge+βAge2+ηYi+σεij,

where IL-6_*ij*_ refers to the IL-6 measurement corrected for cell density. Index “*i*” refers to cell culture ID 1 to 28 and index “*j*” refers to the row *versus* column-plate field within each cell culture, from 1 to 8. *Y*_*i*_ refers to the random effect of cell culture *i*; hence *η* measures the degree of variation between cell cultures, while *σ* is a measure of the variation within the individual cell culture. A similar model was applied to the other cytokines and chemokines studied. The concentrations of cytokines and chemokines were corrected for cell density in the eight-field plates of the individual cell culture through division by the degree of confluence and transformed with the natural logarithm to stabilize the variance. To avoid problems with observations of zero, one was added to all observations before transformation of the data. The model for each cytokine was then reduced by means of the maximum-likelihood method through standard significance testing, and estimates for levels for each combination of treatment and diagnosis were calculated for both stimulated and nonstimulated cells. The levels were estimated through the mean in a log-normal distribution, taking both mean and variance of the transformed data into account. Confidence bands were found as the inverse logarithm of the mean ± 1.96 times the standard deviation for the transformed variables, thus making the confidence bands asymmetric. Likelihood ratio tests were evaluated through the chi-square distribution. 

## 3. Results

In an ideal world we would select cryo-preserved synoviocytes representing a balanced proportion between sex and age of the subjects from whom the cell isolates came. With the possibilities presented to us, the distribution and age (mean, SD, range) of the donor subjects were as follows: OA: males 0, females 12, age (52.2 y, 10.5 y, 33.1–69.1 y); RA: males 2, females 8, age (48.8 y, 13.8 y, 28.2–64.5 y); HC: males 6, females 0, age (33.3 y, 2.3 y, 30.1–36.4 y). The figures for the total population were males 8, females 20, age (46.9 y, 12.6 y, 28.2–69.1 y). The deviation of the distributions from equality is significant, *P* < 0.00005 as to sex and *P* < 0.006 as to age, reflecting that the potential HC donors in the hospital were younger sportsmen.

The localization of the source joints and the type of specimen drawn and cultured, are listed in Table 1.

A summary of the individual compositions of each cell population transferred to eight-well cell-culture plates, and used for a substance exposure experiment, is shown in Table 2. 

Only CD90-positive cells, indicating fibroblast-like synovial cells or activated endothelial cells, constituted a major proportion. Presence of macrophage-like synovial cells, labelled with anti-CD14, as not demonstrated, nor were macrophage-like synovial cells or unstimulated endothelial cells, labelled with anti-CD31, demonstrated. The results referring to labelling with anti-CD68 suggested that up to 2% of the cell population may have been macrophage-like synovial cells. 

The degree of confluence was found constant at visual inspections under the microscope every three days throughout each experiment.

All cell cultures, irrespective of treatment, produced hydroxyproline, which again indicated fibroblast-like cells with their usual production of extracellular substances. There was no dependence on treatment or stimulation. There was a nonsignificant trend towards higher production in OA and RA compared to HC.

Histamin production was measured in all cell cultures but showed no dependence on diagnosis, stimulation, or treatment.

The statistical analysis of the concentrations of cytokines and chemokines revealed no significant triple interaction between diagnosis, stimulation, or no stimulation with TNF-*α*, and drug treatment for any of the cytokines/chemokines measured, and no effect of sample type, localization, sex, or age. Treatment did not interact with diagnosis or stimulation; hence the final model was

(2) $$\begin{array}{c} \;\ln\left(\text{IL-}6_{ij}\right)=\beta_{\text{Diagnosis},\;\text{Stimulation}}+\beta_{T}+\eta Y_{i}+\sigma \varepsilon_{ij}. \end{array}$$

The final model for the other cytokines and chemokines studied as found to be equivalent. 

The dual-interaction between diagnosis and TNF-*α* stimulation was significant as to IL-1*β* (*P* = 0.02), IL-6 (*P* = 0.03), and IL-8 (*P* < 0.0001). 

**Figure 1:**
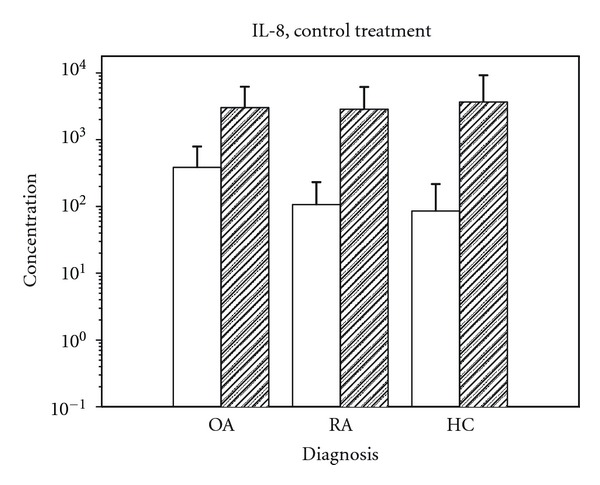
IL-8 production, given in log pg/mL, in cells from OA, RA and HC without stimulation (light columns) and following TNF-*α* stimulation (dark columns).

Fibroblast-like synovial cells *in vitro* in our experiments secreted an increased amount of the pro-inflammatory cytokines IL-1*β*, IL-6, and IL-8 in response to stimulation with TNF-*α*, both untreated and when treated with anti-inflammatory substances. This was the case with cells originating from either OA, RA, or HC individuals, as illustrated for IL-8 in Figure 1. 

The concentrations of IL-6 in the conditioned media from nonstimulated and stimulated cells at the different diagnoses and treatments are shown in Table 3. 

Unstimulated OA cells showed a higher IL-6 production than unstimulated RA and HC cells, which showed a similar IL-6 production. TNF-*α* stimulation caused less increase in IL-6 production in OA cells than in RA or HC cells, which showed similar responses to TNF-*α* stimulation.

Likewise, IL-8 production was higher in un-stimulated cells from OA patients than in cells from RA or HC subjects. Following TNF-*α* stimulation, the IL-8 production, and thereby measured IL-8 concentration, was increased to a similar level irrespective of diagnosis group.

Un-stimulated OA-cells did not differ from un-stimulated RA and HC cells concerning IL-1*β* production. However, following TNF-*α* stimulation, IL-1*β* production increased for all cells measured, but a higher increase in IL-1*β* production was seen in RA cells than in OA cells, which both presented a higher increase than HC cells. 

IL-11, IL-12p70, IL-15, and IL-17 were measured in variable, low concentrations, with several cultures not exceeding the lower level of detection, indicating a general low production of these cytokines and no response in expression of these to TNF-*α* stimulation. CCL2 showed a high increase in production after TNF-*α* stimulation, but this was not significant in the statistical analysis due to a large variation between individual cell cultures. MIF concentrations were relatively high, around 500 pg/mL, and did not change with TNF-*α* stimulation. 

**Figure 2:**
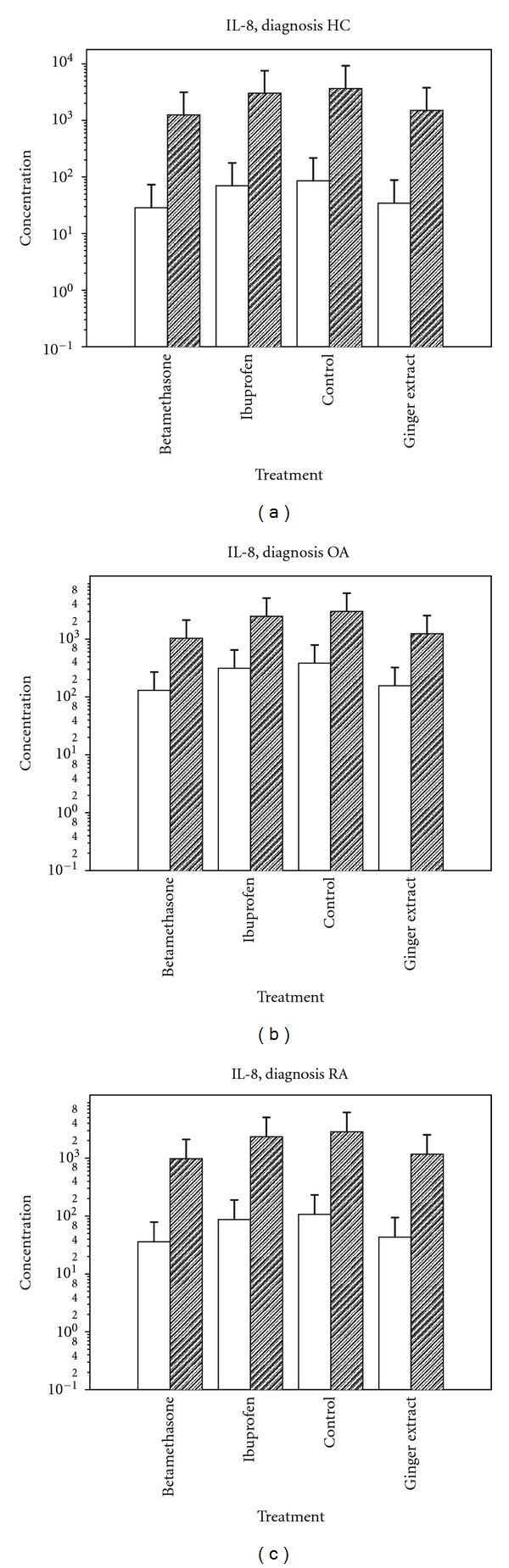
IL-8 production, given in log pg/mL, in cells from HC (a), OA (b), and RA (c), unstimulated (light columns) and stimulated with TNF-*α* (dark columns), when exposed to betamethasone, ibuprofen, no treatment (control), or ginger extract treatment.

When looking at response to the anti-inflammatory drugs, illustrated for IL-8 in Figures 2(a)–2(c) and for IL-6, Table 3, ibuprofen showed no effect, while the ginger extract and betamethasone were both effective and showed a similar reduction of cytokine production. This is also seen from the *P* values when comparing the different treatments, shown in Table 4.

## 4. Discussion

Overall, our study shows that the cell model developed here may be a useful *in vitro* model, both in differentiating between OA and RA, and when testing the effectiveness of various anti-inflammatory drug substances. 

The cell cultures determined to be fibroblast-like synoviocytes were well functioning [24] indicated by their hydroxyproline production throughout the study, as well as by the constant degree of confluence.

Histamine production was found not to be influenced by diagnosis, stimulation, or treatment. This indicates that self-produced histamine was not part of the cell culture's response to stimulation. This behaviour is in line with what is expected from a synoviocyte population [25]. 

Early diagnosis of RA in cases not yet fulfilling the ACR criteria [11] may benefit from looking at differences in IL-6 production from fibroblast-like synoviocyte cell cultures. Since no subject-related dependent variables like age were included in the statistical model, it may be possible to provide a method for distinguishing the cytokine reactions of fibroblast-like synoviocytes isolated from subjects from these groups. A larger cell material from a population of OA and RA patients must though be measured to document that the difference seen in our study is a general difference between cells from these two populations. The fact that the cells in our study came from different joints speaks, on the other hand, for that the fibroblast-like synovial cells studied here have some general features when they respond to TNF-*α* and that OA and RA cells show some real differences here. Our healthy controls had no prehistory of arthritic disease and came to the clinic due to physical trauma or overuse. They showed no sign of inflammatory disease or sign of arthritis in any form. The healthy control fibroblasts in our study must therefore be considered as a good representation of cells from a healthy joint. As with the OA and RA cells, a higher number of subjects would have strengthened the data, but getting permission to take these samples is not easy, and the number in the study is high enough for the statistical methods applied. Overall, our results based on the cell populations studied must be considered reliable, although one must be careful in generalising the results, until more studies are carried out.

The striking effect of the ginger extract on secretion of proinflammatory cytokines, showing as good an effect as betamethasone in reducing production of these cytokines, is a novel finding which may lead to reconsideration of the effect of ginger extract as a possible drug in arthritis therapy. The effect on inhibition of TNF-*α* production by the ginger extract was earlier reported in synoviocytes by Frondoza et al. [26] and Phan et al. [27], and ginger is known to have a moderate effect on osteoarthritis *in vivo* [28, 29]. Funk et al. [30] also found a promising effect of ginger extract in experimental arthritis in rats. Our results are in accordance with these earlier studies and suggest an effect of ginger on inflammatory processes at cell level. Furthermore, we show that ginger in the form of a mixture of substances from both *Zingiber officinale *and *Alpinia galanga,* as applied in our model, is as effective an anti-inflammatory drug as Betamethasone in the population of RA-derived fibroblasts. Use of ginger as an anti-inflammatory could be suggested as a supplement to the anti-inflammatory drugs applied in RA treatment, but clinical studies are needed to support if the results in our study are paralleled to an effect *in vivo*. That ibuprofen in the same situation shows close to no effect on our cells can only be interpreted as ibuprofen's effect must take place at another level of the inflammatory processes, since the solutions applied were made up on a day to day basis, and a dependence on only one batch with an ineffective drug, causing the seen result, could be ruled out. Another interpretation could have been that ibuprofen was applied at too low a concentration. The concentration applied was though as high as it was possible in terms of solubility in aqueous media, ten times lower than saturated. *In vivo*, ibuprofen concentrations in blood are expected to have a substantial effect on pain from 11 to 40 *μ*g/mL [31, 32]. In our study, the ibuprofen concentration in the cell medium was 100 *μ*g/mL; that is, the applied concentration was in excess of the effective plasma concentration *in vivo*. The lack of an effect of ibuprofen can therefore not be due to an inefficient batch of the drug or applying a too low concentration for an effect of the drug.

## 5. Conclusions

In conclusion, ginger extract EV77/15 is as effective an anti-inflammatory agent as betamethasone in this *in vitro* cell model of cultured fibroblast-like synoviocytes. Looking at the cytokine response from TNF-*α* stimulated, cultured fibroblast-like synoviocytes, isolated from subjects with OA or RA patients, promise to be a useful pharmacological model, but clinical studies to support results from such a model are needed. 

## Figures and Tables

**Table 1:** Origin of cells by joint localization and type of specimen (SF for synovial fluid; SM for synovial membrane).

Localization	Diagnosis group		
	OA	RA	HC
Elbow	0	1 (1 SF; 0 SM)	0
Finger or hand	0	3 (2 SF; 1 SM)	0
Hip	3 (0 SF; 3 SM)	2 (0 SF; 2 SM)	0
Knee	9 (5 SF; 4 SM)	4 (3 SF; 1 SM)	6 (0 SF; 6 SM)

Total	12 (5 SF; 7 SM)	10 (6 SF; 4 SM)	6 (0 SF; 6 SM)

**Table 2:** Summary of percentages of cells stained positive with specific antibody to binding site, or nonspecific isotype control, for all 28 cell populations used.

	Mean percentage of all 28 cell populations		Minimum		Maximum	
	Specific CD	Isotype control	Specific CD	Isotype control	Specific CD	Isotype control
CD14+ (macrophage-like synoviocytes)	0.15	0.21	0.00	0.10	0.60	0.30
CD31+ (endothelial cells and macrophage-like synoviocytes)	0.08	0.26	0.00	0.20	0.40	0.60
CD68+ (macrophage-like synoviocytes after intracell staining)	0.56	0.30	0.00	0.20	3.60	1.20
CD90+ (fibroblast-like synoviocytes)	52.19	0.79	5.00	0.00	98.00	2.00

**Table 3:** Mean and confidence interval for IL-6 production in cells unstimulated and stimulated with TNF-*α*. Concentrations in pg/mL.

	Diagnose OA	Diagnose RA	Diagnose N
Betamethasone Not stimulated	25.61 (13.37; 49.02)	16.30 (8.08; 32.86)	19.22 (7.98; 46.32)
Betamethasone Stimulated	32.04 (16.74; 61.34)	48.36 (23.89; 98.07)	44.94 (18.65; 108.27)
Ibuprofen Not stimulated	71.13 (37.18; 136.08)	45.27 (22.46; 91.23)	53.40 (22.18; 128.59)
Ibuprofen Stimulated	89.01 (46.53; 170.29)	134.34 (66.55; 271.22)	124.84 (51.84; 300.61)
Control Not stimulated	75.98 (39.72; 145.37)	48.36 (23.99; 97.46)	57.05 (23.69; 137.37)
Control Stimulated	95.09 (49.70; 181.91)	143.5 (71.08; 289.73)	133.36 (55.38; 321.12)
Ginger Extract Not stimulated	36.05 (18.85; 68.98)	22.94 (11.38; 46.24)	27.07 (11.24; 65.18)
Ginger Extract Stimulated	45.12 (23.58; 86.32)	68.10 (33.73; 137.48)	63.28 (26.28; 152.37)

**Table 4:** *P* values for the comparison of the different treatments on IL-6 an IL-8 production.

	IL-6 production	IL-8 production
Control versus betamethasone	<0.0001	<0.0001
Control versus ibuprofen	0.74	0.42
Control versus ginger extract	0.0003	0.0004
Betamethasone versus ibuprofen	<0.0001	0.0005
Betamethasone versus ginger extract	0.10	0.46
Ibuprofen versus ginger	0.0003	0.006
